# Pulmonary *Mycobacterium fortuitum* Infection Associated With Achalasia Treated With Peroral Endoscopic Myotomy Alone: A Case Report

**DOI:** 10.1002/rcr2.70718

**Published:** 2026-09-17

**Authors:** Hidetaka Masuda, Ryo Torii, Kei Yamasaki, Tatsuya Shingu, Ryosuke Hata, Kazuhiro Yatera

**Affiliations:** ^1^ Department of Respiratory Medicine Wakamatsu Hospital of the University of Occupational and Environmental Health, Japan Kitakyushu Japan; ^2^ Department of Respiratory Medicine University of Occupational and Environmental Health, Japan Kitakyushu Japan

**Keywords:** achalasia, aspiration, *Mycobacterium fortuitum*, nontuberculous mycobacteria, peroral endoscopic myotomy

## Abstract

Pulmonary infection caused by *Mycobacterium fortuitum* is uncommon and often occurs in patients with underlying conditions such as achalasia. We report a 50‐year‐old man with achalasia who developed persistent pulmonary consolidation associated with *M. fortuitum* . His respiratory symptoms fluctuated in parallel with gastrointestinal symptoms, suggesting chronic aspiration. Histopathological findings and sputum culture supported the diagnosis of nontuberculous mycobacterial disease. Peroral endoscopic myotomy (POEM) was performed to treat achalasia, resulting in marked clinical and radiological improvement without antimycobacterial therapy. This case highlights the importance of addressing underlying diseases in the management of pulmonary nontuberculous mycobacterial infection.

## Graphical abstract

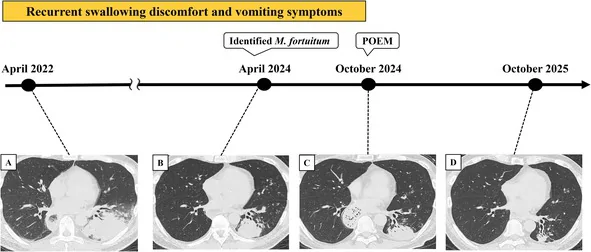

## 1 Introduction

*Mycobacterium fortuitum* is a rapidly growing nontuberculous mycobacterium (NTM) belonging to Runyon group IV, commonly found in environmental sources. It is an uncommon pathogen that can cause infections of the skin and soft tissues, postoperative wounds, catheter‐related infections, and, less frequently, pulmonary disease [1]. Pulmonary infection due to *M. fortuitum* is typically observed as a secondary infection in patients with underlying structural lung diseases or conditions associated with chronic aspiration, including achalasia [2, 3]. The association between achalasia and pulmonary NTM infection has been reported for decades, with rapidly growing mycobacteria frequently implicated [3]. Most previously reported cases have improved with antimycobacterial therapy alone or in combination with treatment for achalasia [2]. However, improvement with treatment of achalasia alone has not been described to date. Herein, we report a rare case of pulmonary *M. fortuitum* infection associated with achalasia that improved following treatment of achalasia alone.

## 2 Case Report

**FIGURE 1:**
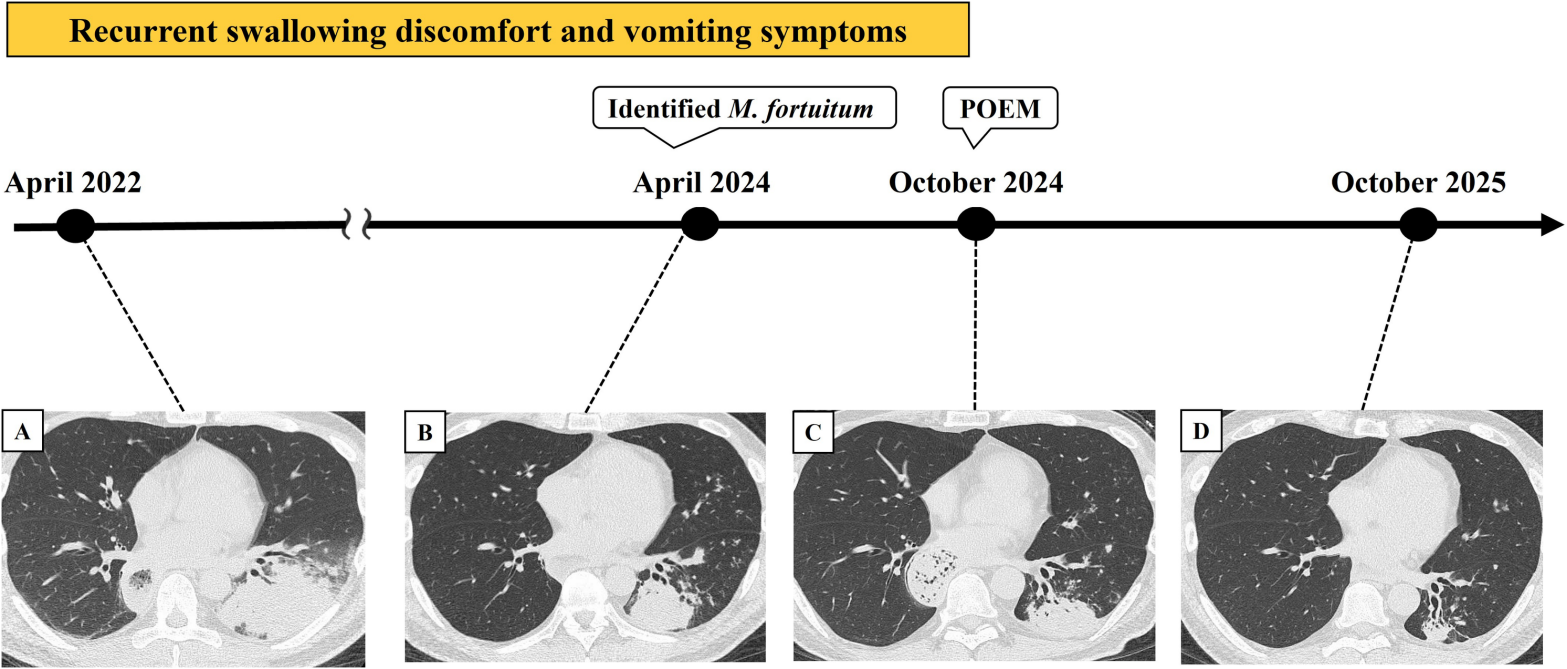
Chest computed tomography findings and clinical course. Chest computed tomography (CT) on admission (April 2022) showed consolidation with ground‐glass opacities in the left lower lobe (A), which persisted despite antibiotic and corticosteroid therapy on follow‐up imaging (B and C) and subsequently improved on follow‐up CT obtained one year after POEM (October 2025) (D); the clinical course showed recurrent swallowing discomfort and vomiting temporally associated with exacerbations of respiratory symptoms, with *Mycobacterium fortuitum* identified in April 2024 and sustained clinical and radiological improvement after POEM.

A 50‐year‐old Japanese man presented with fever and a dry cough in March 2022 and was initially treated with antibiotics for suspected pneumonia; however, his symptoms did not improve, and he was admitted to our hospital in April 2022. Computed tomography (CT) revealed consolidation with ground‐glass opacities in the left lower lobe (Figure 1A). Laboratory findings showed leukocytosis (17,600/μL) and an elevated C‐reactive protein level (15.0 mg/dL) (Table 1). He had a history of oesophageal achalasia and had undergone balloon dilation at 35 years of age. Dysphagia and vomiting had gradually worsened over the previous 5 years despite medical therapy. Sputum culture yielded *M. fortuitum* once; however, it was not isolated from subsequent sputum cultures. Therefore, the findings were not considered sufficient to establish a diagnosis of nontuberculous mycobacterial disease, and the patient was initially diagnosed with aspiration pneumonia and secondary organising pneumonia. He was treated with antibiotics and corticosteroids; however, both clinical symptoms and radiological findings persisted (Figure 1B,C). His respiratory symptoms fluctuated in parallel with gastrointestinal symptoms, worsening during periods of increased vomiting and improving when these symptoms subsided.

**TABLE 1:** Results of peripheral blood analysis on admission.

Blood cell counts		Blood chemistry		Serology	
WBC	17,600/μL	TP	7.8 g/dL	CRP	15.0 mg/dL
Neutrophils	85.8%	Alb	2.8 g/dL		
Lymphocytes	9.3%	T‐bli	0.5 mg/dL	IGRA	Negative
Eosinophils	0.2%	AST	28 IU/L	Anti‐MAC antibody	2.62 U/mL
Monocytes	5.6%	ALT	68 IU/L		
Basophils	0.5%	LDH	164 IU/L		
RBC	367 × 10^4^/μL	γ‐GTP	153 IU/L		
Hb	10.7 g/dL	BUN	14 mg/dL		
Ht	31.5%	Cre	1.2 mg/dL		
Platelets	51.3 × 10^4^/μL				

Abbreviations: γ‐GTP, gamma‐glutamyl transferase; Alb, albumin; ALT, alanine aminotransferase; AST, aspartate aminotransferase; BUN, blood urea nitrogen; Cre, creatinine; CRP, C‐reactive protein; Hb, haemoglobin; Ht, haematocrit; IGRA, interferon gamma releasing assay; LDH, lactate dehydrogenase; MAC, *Mycobacterium avium* complex; RBC, red blood cell; T‐bil, total bilirubin; TP, total protein; WBC, white blood cell.

In April 2024, bronchoscopy was repeated due to symptom exacerbation. Histopathological examination of specimens obtained from the left lower lobe revealed non‐caseating granulomas, and Ziehl–Neelsen staining demonstrated acid‐fast bacilli. Based on these findings, together with the previous positive sputum culture and the clinical course, he was diagnosed with pulmonary nontuberculous mycobacterial disease due to *M. fortuitum* associated with chronic aspiration secondary to achalasia, according to the current Japanese diagnostic criteria (clinical and radiological findings with exclusion of alternative diagnoses, combined with microbiological or histopathological evidence, including a single positive culture with compatible histopathology). Although there was a time interval between the microbiological and histopathological findings, the patient had persistent respiratory symptoms and radiological abnormalities throughout the observation period, and transbronchial lung biopsy demonstrated non‐caseating granulomas with acid‐fast bacilli. These findings supported a persistent disease process rather than transient colonisation or contamination. Given that control of the underlying disease was considered essential, peroral endoscopic myotomy (POEM) was performed for achalasia in October 2024. Following this intervention, his gastrointestinal symptoms improved markedly. Without initiating antimycobacterial therapy, his respiratory symptoms, including cough and fever, resolved, and radiological findings showed marked improvement (Figure 1D). During more than 1 year of follow‐up, no recurrence of pneumonia or NTM‐related disease has been observed, although mild residual fibrosis remains.

## 3 Discussion

This case highlights a rare presentation of pulmonary infection associated with *Mycobacterium fortuitum* in the setting of oesophageal achalasia, which showed marked clinical and radiological improvement following treatment of achalasia alone, without antimycobacterial therapy. The close temporal relationship between gastrointestinal symptoms and respiratory exacerbations strongly supports chronic aspiration as a key pathogenic mechanism.

Achalasia is a primary oesophageal motility disorder characterised by impaired lower oesophageal sphincter relaxation and absent peristalsis, resulting in food stasis and recurrent aspiration [4]. Nontuberculous mycobacteria are commonly ingested with food and may colonise the upper gastrointestinal tract. In patients with achalasia, repeated microaspiration of retained oesophageal contents is considered to play an important role in the development of pulmonary NTM infection [3]. In the present case, respiratory symptoms fluctuated in parallel with vomiting episodes, further supporting this mechanism. In addition, lipid‐rich conditions within retained oesophageal contents may contribute to the pathogenicity of rapidly growing mycobacteria such as *M. fortuitum* . Previous studies have suggested that lipid environments can impair macrophage phagocytosis and promote the growth of mycobacteria [5]. In this case, dietary habits favouring high‐fat intake may have facilitated bacterial persistence and infection, although this remains speculative.

A diagnostic challenge in this case was the interval between the microbiological and histopathological findings. Although the microbiological evidence alone would have been insufficient according to international diagnostic criteria, transient colonisation or contamination was considered unlikely because the patient had persistent respiratory symptoms and radiological abnormalities over 2 years, respiratory exacerbations repeatedly paralleled worsening achalasia symptoms, and transbronchial lung biopsy demonstrated non‐caseating granulomas with acid‐fast bacilli. Together with the previous positive sputum culture, these findings fulfilled the current Japanese diagnostic criteria for pulmonary nontuberculous mycobacterial disease. Nevertheless, the interval between the microbiological and histopathological findings should be recognised as a limitation when interpreting this case.

Several cases of pulmonary nontuberculous mycobacterial infection associated with achalasia have previously been reported. As summarised in Table 2 [2, 6, 7, 8, 9, 10, 11, 12, 13, 14], most patients received antimycobacterial therapy in combination with treatment for the underlying oesophageal disorder, although the causative mycobacterial species and therapeutic approaches varied. In contrast, our patient achieved sustained clinical and radiological improvement following POEM alone without antimycobacterial therapy. To our knowledge, this appears to be the first reported case demonstrating successful disease control after treatment of achalasia alone. These findings suggest that elimination of chronic aspiration may have contributed to disease control by restoring effective host defence mechanisms. However, because this is a single observational case, causality cannot be established. Although the temporal relationship between POEM and sustained clinical improvement supports this hypothesis, spontaneous fluctuation of disease activity or delayed effects of previous treatments cannot be completely excluded. Another limitation of this case is the lack of microbiological follow‐up after POEM. Because the patient's respiratory symptoms resolved completely, he was no longer able to expectorate sputum, and follow‐up sputum cultures could not be obtained. Therefore, microbiological eradication could not be confirmed.

**TABLE 2:** Reported cases of pulmonary nontuberculous mycobacterial infection associated with achalasia.

Year	Age/sex	Common symptoms	Mycobacterial species	Treatment for mycobacteria	Endoscopic or surgical treatment for achalasia	Outcome	References
2004	59/M	Fever/Wet cough/Dyspnoea	*M. fortuitum*	AMK, IPM and CAM → CPFX, IPM and CAM	None	Improved	[6]
2006	51/F	Cough/Weight loss/Anorexia/Dysphagia/Vomiting	*M. fortuitum*	CPFX and CAM	PD	Improved	[7]
2007	37/M	Fever/Dry cough/Weight loss/Weakness	*M. abscessus*	CAM and MINO	PD	Improved	[8]
2016	11/M	Fever/Wet cough	*M. abscessus*	IPM, AMK and CAM → DOX, CPFX and CAM	Heller myotomy	Improved	[9]
2016	36/M	Fever/Cough/Chills/Dysphagia	*M. abscessus*	TGC, LZD and AMK	Heller myotomy and fundoplication	Improved	[10]
2018	15/F	Fever/Wet cough/Fatigue/Loss of appetite/Vomiting	*M. fortuitum*	TMP‐SMX, CPFX and CAM	Heller myotomy	Improved	[2]
2024	51/M	Fever/Dyspnoea/Fatigue/Myalgia	*M. thermoresistibile*	AMK and MFLX → LZD, MFLX, DOX, TMP‐SMX and AZM	PD → POEM	Improved	[11]
2024	34/M	Cough/Dyspnoea/Fatigue/Weight loss	*M. wolinskyi*	DOX, TMP‐SMX and CPFX	PD	Improved	[12]
2025	51/M	Fever/Cough/Dyspnea/Lethargy	*M. thermoresistibile*	AMK, MFLX, AZM, RFP and EB	PD → POEM	Improved	[13]
2025	59/M	Dyspnoea	*M. fortuitum*	AMK, IPM and LVFX → DOX and TMP‐SMX	None	Ongoing treatment	[14]
Present case	50/M	Fever/Dry cough	*M. fortuitum*	None	PD → POEM	Improved	

Abbreviations: AMK, amikacin; AZM, azithromycin; CAM, clarithromycin; DOX, doxycycline; EB, ethambutol; IPM, imipenem; LVFX, levofloxacin; LZD, linezolid; MFLX, moxifloxacin; MINO, minocycline; PD, pneumatic dilatation; POEM, peroral endoscopic myotomy; RFP, rifampicin; TGC, tigecycline; TMP‐SMX, trimethoprim‐sulfamethoxazole.

In conclusion, clinicians should consider oesophageal achalasia as an underlying cause of pulmonary NTM infection, particularly in patients with fluctuating respiratory symptoms. This case underscores the importance of addressing the underlying disease, as treatment of achalasia alone may contribute to infection control and prevention of recurrence.

## Author Contributions

H.M. and R.T. wrote the draft of the manuscript. K.Y., T.S., R.H. and K.Y. revised it critically. All authors read and approved the final manuscript.

## Consent

The authors declare that written informed consent was obtained for the publication of this manuscript and accompanying images using the consent form provided by the Journal.

## Conflicts of Interest

The authors declare no conflicts of interest.

## Data Availability

Data sharing not applicable to this article as no datasets were generated or analysed during the current study.
